# Functional Analysis of *CLE26* in Controlling *De Novo* Root Regeneration from Detached *Arabidopsis* Leaves

**DOI:** 10.3390/ijms252313156

**Published:** 2024-12-07

**Authors:** Geng Zhang, Yuxuan Du, Xinying Wang, Yuge Zhang, Shili Zhang, Mingyang Li, Xiaojuan Li, Guifang Zhang

**Affiliations:** 1 State Key Laboratory of Efficient Production of Forest Resources, College of Biological Sciences and Technology, Beijing Forestry University, Beijing 100083, China; 2 Key Laboratory of Genetics and Breeding in Forest Trees and Ornamental Plants, Ministry of Education, Beijing Forestry University, Beijing 100083, China; 3 State Key Laboratory of Tree Genetics and Breeding, College of Biological Sciences and Technology, Beijing Forestry University, Beijing 100083, China

**Keywords:** *de novo* root regeneration, *Arabidopsis*, leaf explants, adventitious root, *CLE26*, reactive oxygen species, salicylic acid

## Abstract

*De novo* root regeneration is the process by which adventitious roots form around the wound site from wounded or detached plant organs. The *de novo* root regeneration process has been widely exploited in cutting technology used for vegetative propagation. Here, we employed detached leaf explants from *Arabidopsis thaliana* to form adventitious roots for studying the process of *de novo* root regeneration. GUS staining showed that the expression of *CLAVATA3/EMBRYO SURROUNDING REGION-RELATED26*(*CLE26*) was gradually increased surrounding the wound site of leaf explants during adventitious root formation. Semi-thin sections further showed that the expression pattern of *CLE26* was closely linked to the formation of adventitious roots. Next, genetic analyses confirmed that the *CLE26* gene was involved in *de novo* root regeneration. Furthermore, RNA sequencing (RNA-seq) of the leaf explants revealed that stress-related genes might be involved in *CLE26*-mediated adventitious root formation. Specifically, genes associated with the hydrogen peroxide catabolic process and oxidative stress response were predominantly upregulated in the *cle26* mutant. In contrast, genes involved in the response to salicylic acid were largely downregulated in the *cle26* mutant. Overall, our study indicates that the mutation in *CLE26* might upregulate the expression of genes involved in reactive oxygen species metabolism or suppress the expression of genes associated with salicylic acid synthesis, thus promoting the formation of adventitious roots. These findings suggest that *CLE26* is a potential candidate for the genetic improvement of adventitious rooting in cuttings.

## 1. Introduction

*De novo* root regeneration is a common phenomenon in many plant species when detached organs encounter suitable conditions [1,2,3,4,5,6]. This process refers to the formation of adventitious roots from wounded or detached organs [7,8,9], which has been widely exploited in horticulture, agriculture, and forestry for a long time, such as cuttings. *De novo* root regeneration can be divided into three phases [10,11]. In Phase I, wounding, environmental, and developmental signals induce rapid auxin biosynthesis. In Phase II, auxin production in mesophyll cells transports to regeneration-competent cells (procambium or its nearby parenchyma cell in leaf explants). In phase III, auxin triggers the cell fate transition of the regeneration-competent cells to form adventitious roots [10,11]. The first-step cell fate transition is from regeneration-competent cells to root founder cells [9]. The second-step cell fate transition involves root founder cells dividing to form root primordium [7,11].

The regenerative capacities of plants are highly dependent on the totipotency or pluripotency of somatic cells, whose fates are influenced by phytohormones, wounding, and other stimuli [6,8,11,12]. Recent studies have revealed that the CLAVATA3/EMBRYO SURROUNDING REGION-RELATED (CLE) peptides play a crucial role in regulating the maintenance and differentiation of plant stem cells [13,14,15,16]. Among them, CLAVATA3(CLV3), the member of the CLE peptide family, plays a vital role as a mobile signal in regulating the size of the shoot apical meristem in *Arabidopsis thaliana* [17]. Additionally, CLE peptides are also implicated in the development and formation of the root apical meristem. Specifically, *CLE40*, highly expressed in differentiated columella cells and stele, suppresses (*WUSCHEL-RELATED HOMEOBOX 5*) *WOX5* expression through the receptor-like kinases ARABIDOPSIS CRINKLY4 (ACR4) and CLV1 [18]. Furthermore, CLE9/10 peptides bind to (BARELY ANY MERISTEM) BAM-like receptor kinases to inhibit the periclinal division of xylem precursor cells, thereby regulating root xylem development [19]. More recent studies have also indicated that CLE signaling pathways are intricately involved in organizing tissue layer formation in the *Arabidopsis* root meristem [20].

Intriguingly, CLE26 has emerged as a key regulator in modulating root growth. Previous reports have demonstrated that AtCLE26 application is able to induce a root architectural change and increase the density of the lateral root [21,22]. In addition, *CLE26* can be activated by Dof-class transcription factors and subsequently induce the heterodimerization of BAM1/3 and CLV3 INSENSITIVE KINASEs (CIKs) to suppress the protophloem differentiation during root formation [23,24]. Consequently, we hypothesize that CLE26 might play a crucial role in the cellular reprogramming involved in *de novo* root regeneration.

Here, we focused on the CLE26 peptide in *Arabidopsis*. We conducted genetic experiments to elucidate the role of *CLE26* in adventitious root formation. Furthermore, we identified downstream genes potentially regulated by *CLE26* through transcriptome analysis. This research will significantly enhance our understanding of the mechanisms of CLE peptides in *de novo* root regeneration and lay the foundation for improving cutting propagation through molecular techniques.

## 2. Results

### 2.1. The Expression of CLE26 Is Closely Linked to the Formation of Adventitious Root Primordia

To clarify the role of *CLE26* in *de novo* root regeneration, we exploited adventitious rooting systems in *Arabidopsis*, in which adventitious roots can regenerate from the wounded site on the leaf explants without added hormones [9,25]. We first obtained *CLE26_pro_*: *GUS* reporter lines, in which the promoter of *CLE26* was fused to the gene encoding GUS. The expression patterns of *CLE26* in the primary root and lateral roots are consistent with previous reports (Figure S1) [21]; thus, the *CLE26_pro_: GUS* reporter lines can be used for further analyses.

**Figure 1:**
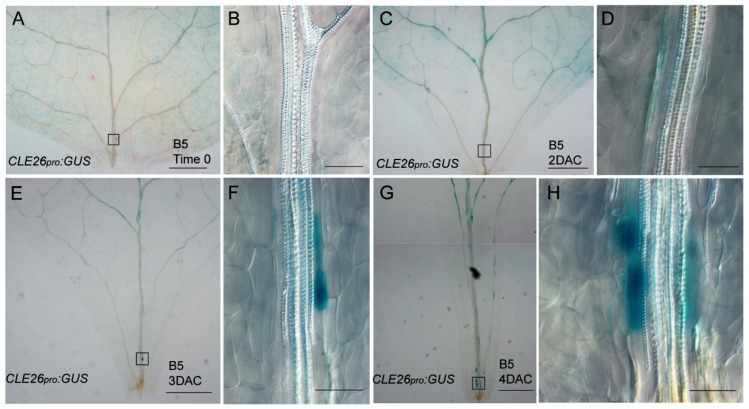
Expression patterns of *CLE26* during *de novo* adventitious root regeneration. GUS staining of *CLE26_pro_*: *GUS* in leaf explants at time0 (**A**,**B**), 2DAC (**C**,**D**), 3DAC (**E**,**F**), and 4DAC(**G**,**H**) cultured on B5 medium. *n* > 10 (**A**–**H**). Bars = 500 μm in (**A**,**C**,**E**,**G**) and 50 μm in (**B**,**D**,**F**,**H**).

We then analyzed the expression patterns of *CLE26* in adventitious rooting from leaf explants using *CLE26_pro_*: *GUS*. No GUS signal was detected in vascular tissues near wounds in time-0 explants (Figure 1A,B) and barely in 2-DAC leaf explants (Figure 1C,D). The expression of *CLE26* could be observed during the initial cell divisions at 3 DAC (Figure 1E,F) and gradually increased near the adventitious root primordium as it developed (Figure 1G,H). To better understand the *CLE26* expression pattern, we then analyzed transverse sections of *CLE26_pro_*: *GUS* leaf explants. GUS staining was mainly concentrated in the procambium cells and the vascular parenchyma cells (Figure S2), which are regeneration-competent cells. These results indicate that *CLE26* may be involved in the formation of the adventitious root primordium during *de novo* root regeneration.

### 2.2. CLE26 Contributes to Adventitious Root Formation

#### 2.2.1. The Mutation in CLE26 Can Enhance the Regeneration of Adventitious Root

**Figure 2:**
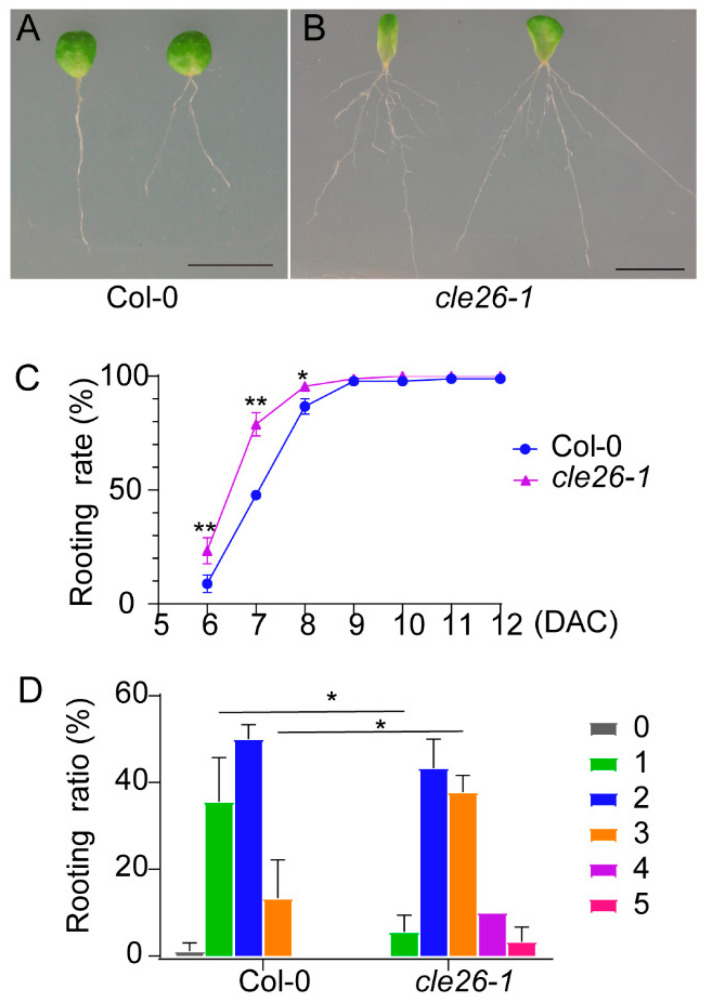
*CLE26* mutation promotes the capacity of adventitious rooting. (**A**,**B**) Leaf explants from wild-type Col-0 (**A**) and *cle26-1* mutant (**B**) at 14 DAC on B5 medium. (**C**) Rooting rate of leaf explants (percentage of leaf explants with regenerated adventitious roots) from Col-0 and *cle26-1*. (**D**) Quantitative analyses of the adventitious root number per 14-DAC leaf explant from Col-0 and *cle26-1* on B5 medium. Bars in C and D show SD from three biological repeats (*n* = 30 per repeat). * *p* < 0.05 and ** *p* < 0.01 in two-tailed Student’s *t*-tests compared with Col-0 (**C**,**D**). Bars = 1 cm (**A**,**B**).

To better understand the role of *CLE26* in adventitious root formation, we analyzed the phenotype of adventitious roots formed from leaf explants of the *cle26-1* mutant (Figure 2A,B). Compared with that in wild-type Columbia0 (Col-0), the rooting was significantly accelerated in leaf explants of the *cle26-1* mutant (Figure 2C), suggesting that mutations in *CLE26* can promote the development of adventitious roots. In addition, the *cle26-1* leaf explants produced more adventitious roots than the wild-type Col-0 (Figure 2D). The number of adventitious roots produced per leaf explant from the wild-type Col-0 was mainly one or two, while that from the *cle26* mutant was mainly two or three, even up to four or five (Figure 2D). These data suggest that *CLE26* is involved in adventitious root formation.

#### 2.2.2. CLE26 Peptide Inhibits the Formation of Adventitious Root

**Figure 3:**
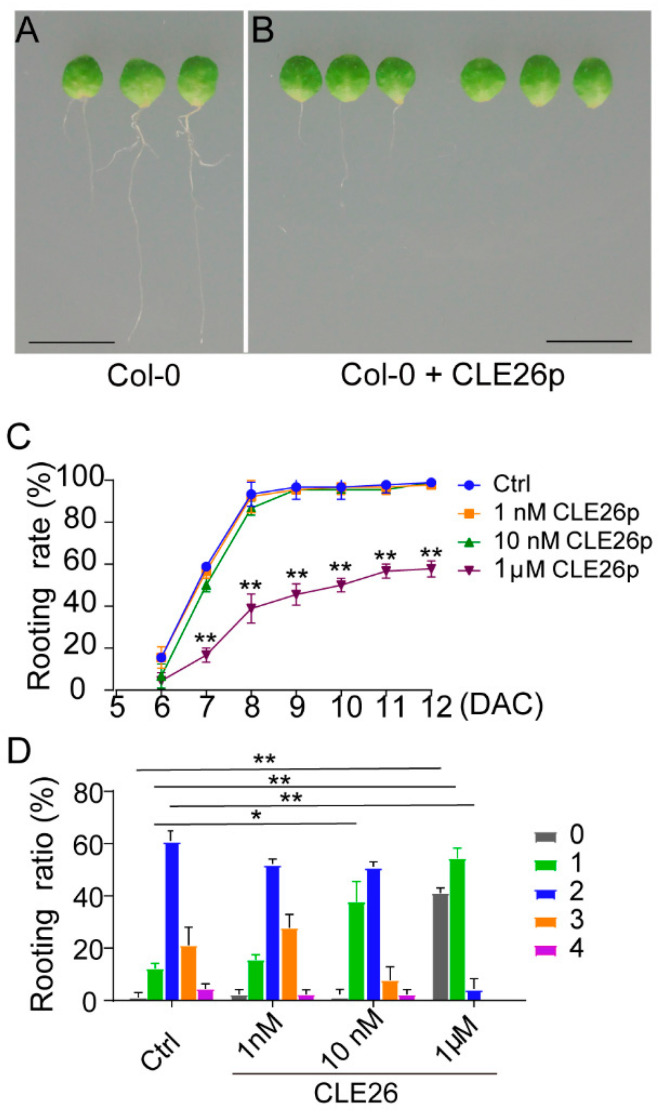
CLE26 peptide inhibits the formation of adventitious roots. (**A**,**B**) Leaf explants from Col-0 at 14 DAC on B5 medium (**A**) and B5 medium with 1 μM CLE26 peptide (**B**). (**C**) The rooting rate of leaf explants on B5 medium containing 0 nM (Ctrl), 1 nM, 10 nM, and 1 μM CLE26 peptide. (**D**) The ratio of adventitious root number per 14-DAC leaf explant on B5 medium (Ctrl) 1 nM,10 nM, and 1 μM CLE26 peptide. Bars in G and H show SD from three biological repeats (*n* = 30 per repeat). * *p* < 0.05 and ** *p* < 0.01 in two-tailed Student’s *t*-tests compared with mock (**C**,**D**). Bars = 1 cm (**A**,**B**).

To further confirm the involvement of *CLE26* in *de novo* root regeneration, a chemically synthesized CLE26 peptide (CLE26p) was used in the wild type. Intriguingly, the rooting process was affected by CLE26p (Figure 3A,B). A dose–response analysis showed that the rooting time was slightly delayed by CLE26p at a concentration of 10 nM and strongly delayed at a concentration of 1 μM (Figure 3C). Similarly, the number of adventitious roots that regenerated from leaf explants treated with CLE26p was mildly reduced at a concentration of 10 nM, and this number was significantly decreased at a concentration of 1 μM (Figure 3D). Therefore, synthetic CLE26 peptide impaired adventitious root development.

The results of these phenotype analyses indicate that *CLE26* acts as a negative regulator in the process of *de novo* root regeneration.

### 2.3. Transcriptional Profiling Identifies Differentially Expressed Genes

**Figure 4:**
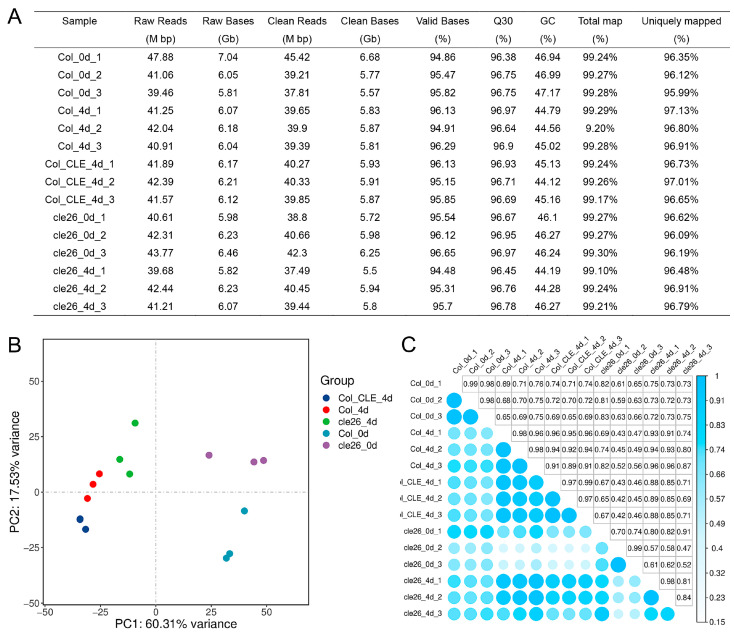
Overview of the transcriptome data. (**A**) Transcriptome assembly and annotated statistics. (**B**) PCA among 15 samples. (**C**) Pearson correlation between samples analysis.

To investigate the molecular mechanism of *CLE26* involved in adventitious root formation, we performed RNA-seq using five samples (Col_0/4 d, cle26_0/4 d, and CLE_4 d). Each sample contained three biological replicates. Subsequently, we constructed fifteen cDNA libraries of these samples for Illumina high-throughput sequencing. After discarding the low-quality reads, approximately 88.43 Gb clean data were produced, yielding a range of 5.5~6.68 Gb clean bases per sample. The Q30 percentage (sequencing error rate < 0.1) was above 96.38%. When mapping the clean reads to the *Arabidopsis thaliana* reference genome, 99.1~99.3% of the reads could be mapped (Figure 4A). According to the principal component analysis (PCA), the three biological replicates of Col_0/4 d, cle26_0/4 d, and CLE_4 d groups clustered separately (Figure 4B). Pearson’s correlation coefficient among the three replicates demonstrates a strong positive correlation (Figure 4C). These results indicate that the data of transcriptome sequencing was reliable.

**Figure 5:**
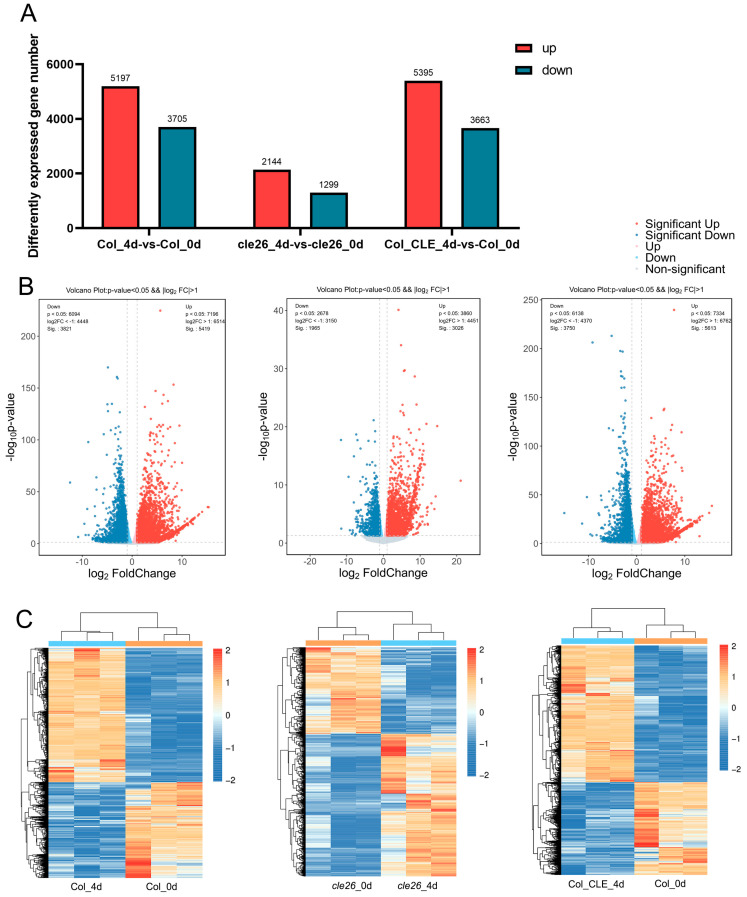
Differentially expressed gene statistics. (**A**) The number of upregulated and downregulated genes in pairwise comparisons. (**B**) Volcano plot showing up-regulated and down-regulated genes in pairwise comparisons. (**C**) Heat maps of upregulated and downregulated genes in pairwise comparisons.

To clarify patterns of gene expression, we identified differentially expressed genes (DEGs) using the criteria |Log_2_(fold-change)| > 1 and *P_adj_* < 0.05 through pairwise comparisons. A total of 8902, 3443, and 9058 DEGs were observed in Col_4d vs. Col_0d cle26_4d vs. cle26_0d, and Col_CLE_4d vs. Col_0d respectively (Figure 5A, Supplementary Table S1). The volcano map showed the gene expression profiles of the DEGs in comparison groups. Compared with Col_0d, 8902 DEGs were produced at Col_4d, of which 5197 genes were upregulated, and 3705 genes were downregulated. Compared with cle26_0d, at cle26_4d, 3443 DEGs were produced, of which 2144 genes were upregulated, and 1299 genes were downregulated. Similarly, compared with Col_0d, 9058 DEGs were produced at Col_CLE_4d, including 5395 upregulated genes and 3663 downregulated genes. The number of upregulated DEGs was greater than the number of downregulated DEGs. We used a heat map to visually display the DEG differences between the comparison groups, and the results showed that the expression patterns were clear between different groups (Figure 5C).

### 2.4. Functional Enrichment Analysis Reveals Key Genes Regulated by CLE26 in Adventitious Root Formation

To better understand the function of these differentially expressed genes obtained from the above analysis, we performed Gene Ontology (GO) enrichment analysis (Figure S3, Supplementary Table S2). Our findings revealed that DEGs in the Col_4d vs. Col_0d mainly participated in response to salicylic acid (SA), leaf senescence, and photosynthesis. In the cle26_4d vs. cle26_0d comparison, DEGs are mainly enriched in protein complex oligomerization, hydrogen peroxide catabolic process, response to oxidative stress, and response to heat. DEGs in the Col_CLE_4d vs. Col_0d are mainly enriched in response to salicylic acid, sulfate assimilation, and cellular response to hypoxia. These results suggest that stress-related genes might be involved in triggering the process of adventitious root formation.

**Figure 6:**
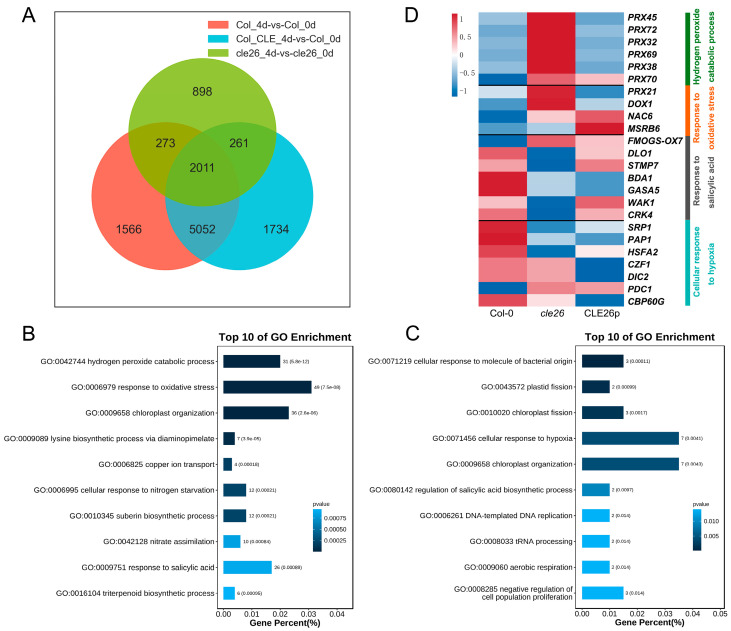
Functional enrichment analysis and characterization of stimulus-responsive genes. (**A**) Venn diagram illustrating the number of DEGs in three pairwise comparisons. (**B**) GO enrichment analysis of the 2011 common DEGs. (**C**) GO enrichment analysis of the 261 common DEGs. Only selected GO terms in the category of biological processes are shown. (**D**) Heatmap analysis of the expression changes in Col-0, *cle26,* and CLE26p over the course of 4 days.

Among the DEGs identified above, 2011 DEGs were common in the three comparisons. Additionally, there were 261 DEGs shared between the cle26_4d vs. cle26_0d and Col_CLE_4d vs. Col_0d comparisons (Figure 6A, Supplementary Table S3). To study the function of these common DEGs, we performed a GO enrichment analysis. The results showed that the 2011 common DEGs were enriched in biological processes related to the hydrogen peroxide catabolic process, response to oxidative stress, and response to salicylic acid (Figure 6B). Moreover, the 261 DEGs were also involved in biological processes related to the cellular response to hypoxia and regulation of the salicylic acid biosynthetic process (Figure 6C). These results reveal that oxygen metabolism and salicylic acid may be involved in *CLE26*-mediated adventitious root formation. To clearly illustrate the differences in gene expression among Col-0, *cle26*, and CLE26p on day 4, we employed a heatmap to represent their expression patterns (Figure 6D). Notably, the heat map analysis showed that the genes participating in the hydrogen peroxide catabolic process and response to oxidative stress were mainly upregulated in the *cle26* mutant, whereas the genes involved in response to salicylic acid and cellular response to hypoxia were predominantly downregulated. Our results indicate that the *cle26* mutant may facilitate adventitious root formation by upregulating the expression of genes implicated in the hydrogen peroxide catabolic process and response to oxidative stress and downregulating the expression of genes linked to salicylic acid and hypoxia.

## 3. Discussion

The CLE family was revealed to play a crucial role in cell reprogramming, including regulation of the shoot apical meristem, root apical meristem, and lateral meristem [19,21,24,26,27,28], but very little is known about their role in *de novo* root regeneration. This study focused on the *CLE26* gene to investigate its functional role in adventitious rooting.

*CLE* expression can be triggered by environmental stimuli [16,26,29]. GUS staining showed that *CLE26* was a response to the wound of leaf explants and was present in the vascular tissues around the wound in *de novo* root regeneration (Figure 1E,G). Previous studies showed that *CLE26* was expressed in the vasculature and pericycle, which contributes to the lateral root patterning [21]. Our analysis indicated that *CLE26* was mainly expressed in the procambium cells and the vascular parenchyma cells. Intriguingly, the appearance of *CLE26* coincided with the development of adventitious root primordia. *CLE26* expression was predominantly localized around the root primordia (Figure 1H), hinting that *CLE26* may play a role in adventitious root patterning by modulating the microenvironment surrounding the adventitious root primordia.

CLE peptides (CLE25/26/45) can facilitate the heterodimerization of BAM and CLV3 INSENSITIVE KINASEs (CIKs), which subsequently leads to the activation of the receptor complex through phosphorylation [23]. This process suppresses the differentiation of the protophloem in the primary root. Our studies showed that the *cle26-1* mutant exhibited a significant capacity for adventitious rooting compared to Col-0(Figure 2). The leaf explants treated with CLE26 peptide showed a deficit in adventitious rooting (Figure 3). The number of adventitious roots from leaf explants treated with CLE26p was reduced at a concentration of 10 nM, a physiologically relevant concentration [21]. The phenotype of the adventitious root was more significant at a concentration of 1 μM, which was consistent with the effect degree on *Arabidopsis* root architecture [21]. Therefore, *CLE26* plays an important role in regulating cell reprogramming of root development. The formation of adventitious roots at the bases of cuttings is a key determinant for vegetative propagation in agricultural technologies [10]. *CLE26* can be modified by gene editing to regulate the regeneration of adventitious roots in cuttings. Additionally, a recent study highlighted the intersection of distinct CLE peptide pathway activities in influencing tissue patterning within the root meristem [20]. The crosstalk of CLE26 peptide with other CLE peptides in controlling *de novo* root regeneration deserves further investigation.

To elucidate the molecular mechanism of *CLE26* in regulating root regeneration, we performed a GO enrichment analysis on the DEGs and found significant enrichment in stress-related signals, notably SA responses and oxidative stress management, highlighting their pivotal roles in *CLE26*-driven root regeneration. Additionally, we identified 2011 genes that were commonly differentially expressed across all three comparison groups (Col_4d vs. Col_0d, cle26_4d vs. cle26_0d, and Col_CLE_4d vs. Col_0d Furthermore, 261 genes were specifically shared between the cle26_4d vs. cle26_0d and Col_CLE_4d vs. Col_0d comparisons. GO enrichment analysis of these common DEGs revealed their primary involvement in hydrogen peroxide catabolic processes, oxidative stress responses, and salicylic acid signaling. The heatmap analysis disclosed that genes associated with the hydrogen peroxide catabolic process and oxidative stress response were significantly upregulated in the *cle26* mutant. It has been reported that appropriate accumulation of reactive oxygen species (ROS) promotes *de novo* root regeneration from leaf explants [30]. More recent evidence indicates that superoxide anions are required for maintaining stem cell fate [31]. In addition, respiratory burst oxidase homolog (RBOHB) regulates non-embryonic callus formation, whose cell identity is the root primordium or root apical meristem [32,33] via catalyzing the production of hydrogen peroxide (H_2_O_2_) [34]. Thus, the *cle26* mutant may facilitate *de novo* root regeneration by enhancing the expression of ROS-related genes.

Furthermore, our results indicated that genes associated with salicylic acid responses and cellular hypoxic responses were downregulated in the *cle26* mutant. A recent study showed that hypoxia-induced RELATED TO APETALA 2.12 (RAP2.12) protein could directly activate the salicylic acid biosynthetic gene *SID2*, promoting salicylic acid accumulation [35]. The accumulation of salicylic acid inhibits the formation of calli, which possess the root identity. Therefore, the *cle26* mutant might promote *de novo* root regeneration by suppressing the expression of genes involved in salicylic acid synthesis. Moreover, salicylic acid inhibited the expression of *CLE26* [21]. Our research findings indicate that salicylic acid-related genes are highly expressed in the Col-0. There may be a feedback regulation between salicylic acid and *CLE26* in the Col-0, which maintains normal root development.

## 4. Materials and Methods

### 4.1. Plant Materials and Culture Conditions

To produce *CLE26pro: GUS* transgenic plants, a 2.27-kb *CLE26* promoter was PCR amplified and inserted into the pBI101 vector using the following primers: 5′-cgggatccAAGCTTTCATCTGCTCACT-3′ and 5′-tcccccgggGGTTTCTAGCCTTTGTGGATA-3′. Transgenic plants were obtained via the *Agrobacterium tumefaciens*-mediated transformation of the construct into Col-0. The *cle26-1*mutant (N689781) was described previously [21]. An unmodified CLE26 peptide (RKVPRGPDPIHN) was ordered from Beijing Tsingke Biotech Co., Ltd. (Beijing, China). and dissolved in sterile water.

For the regeneration of adventitious roots, *Arabidopsis thaliana* seeds were germinated on a half-strength Murashige and Skoog (MS) medium at 22 °C under a 16 h light/8 h dark photoperiod. Detached leaf explants from 12-day-old seedlings were cultured on B5 medium or B5 medium with CLE26p at 22 °C in the dark to induce adventitious roots. The 1/2 MS medium and B5 medium conditions were described previously [7].

### 4.2. GUS Staining and Microscopy

GUS staining was performed by incubation of leaf explants from the *CLE26pro: GUS* transgenic lines at 37 °C in GUS assay solution (5 mM Na_2_EDTA, 2 mM K_3_Fe(CN)_6_, 2 mM K_4_Fe(CN)_6_, 50 mM sodium phosphate buffer pH 7, 0.1% Triton X-100, and 0.04% X-Gluc). The stained tissues were first decolorized in 75% alcohol and then incubated in the chloral hydrate solution (200 g chloral hydrate, 20 g glycerol, and 50 mL water) at 65 °C for 6 h for tissue transparency. Differential interference contrast observations were performed as described previously [36].

### 4.3. RNA-Seq and Transcripts Annotation

For RNA-seq analysis, leaf explants from 12-day-old seedlings of Col-0 and the *cle26-1* mutant were cultured on B5 medium for 4 days, and leaf explants from 12-day-old seedlings of Col-0 were cultured on B5 medium with CLE26 peptide (CLE26p) for 4 days. Leaf explants were used for RNA extraction.

RNA sequencing was conducted in OE Biotech. Co., Ltd. (Shanghai, China). The strand-specific libraries and small RNA sequencing libraries were performed on an Illumina Novaseq 6000 platform (Illumina, San Diego, California, USA). The raw reads in fastq format were initially processed with fastp to remove low-quality reads. Using the HISAT2, clean reads were mapped to the *Arabidopsis thaliana* reference genome (TAIR10.1). FPKM (fragments per kilobase of transcript per million fragments mapped) values for the genes were obtained for each sample. The differentially expressed genes were analyzed using DESeq2 with Fold Change > 2 and *P_adj_* value < 0.05. GO and KEGG pathway enrichment analysis of genes were performed to screen the significantly enriched term using R (v 3.2.0), respectively. Bioinformatic analysis was performed using the OE Cloud tools at https://cloud.oebiotech.com/task/ (accessed on 4 November 2024).

### 4.4. Accession Numbers

The sequence data from this article can be found in the Arabidopsis Genome Initiative, and the accession numbers are listed in Supplementary Table S4.

## 5. Conclusions

CLE peptides play a crucial role in modulating cell reprogramming. This study reveals that CLE26 contributes to *de novo* root regeneration in *Arabidopsis thaliana*. We demonstrated that the mutation in *CLE26* significantly enhanced adventitious root formation, while the application of synthetic CLE26 peptides inhibited this process. Through transcriptome analysis, we observed that the *cle26* mutant exhibited heightened expression of oxidative stress-related genes and a reduction in the expression of genes responsive to salicylic acid, which might contribute to enhanced adventitious root formation. These findings not only provide new insights into the regulatory mechanisms of CLE peptides in root regeneration but also offer a potential application basis for improving cutting propagation through molecular techniques. Future work could explore the function of *CLE26* in woody plants and further elucidate the interplay between *CLE26* and stress-signaling pathways in *de novo* root regeneration.

## Data Availability

The raw data supporting the conclusions of this article will be available from the authors upon request.

## Supplementary Materials

The following supporting information can be downloaded at https://www.mdpi.com/article/10.3390/ijms252313156/s1.
