# Supplementary material for: Functional Analysis of CLE26 in Controlling De Novo Root Regeneration from Detached Arabidopsis Leaves
Source: Int J Mol Sci. 2024 Dec 7;25(23):13156. doi: 10.3390/ijms252313156 (PMC11642536; doi:10.3390/ijms252313156)
Supplement: Supplementary file 1 [file ijms-25-13156-s001.zip › Supplementary figures.pdf]

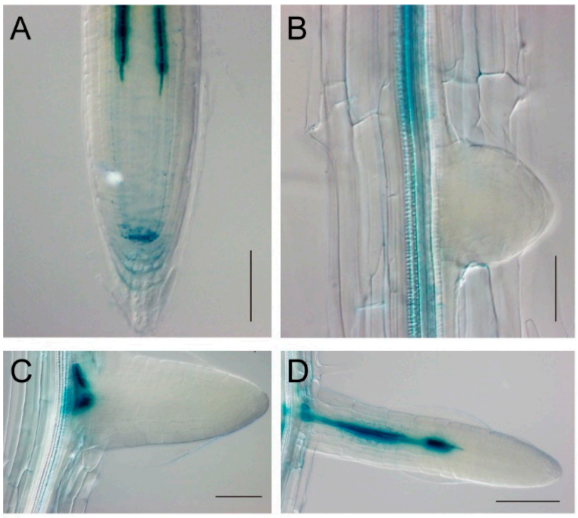

Figure S1. *CLE26* in primary root and lateral root development. (A) Primary root tip from seedlings of $CLE26_{pro}$: *GUS* grown on 1/2 MS medium. (B, C, D) Lateral root meristem in seedlings of $CLE26_{pro}$: *GUS* grown on 1/2 MS medium. Scale bars, 50 μm in (A–D).

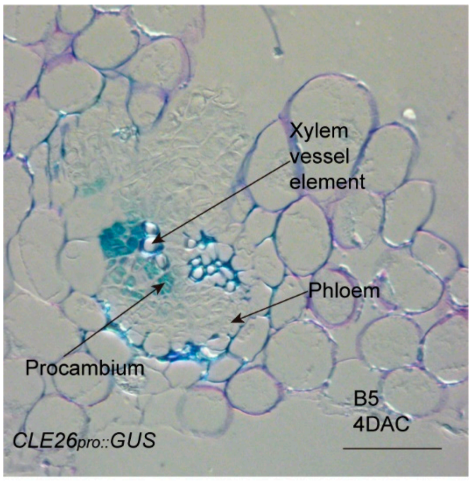

Figure S2 Transverse section through the GUS-staining region of a 4-DAC $CLE26_{pro}$: GUS leaf explant grown on B5 medium. Note that GUS staining was mainly observed in the procambium and the nearby parenchyma cells.

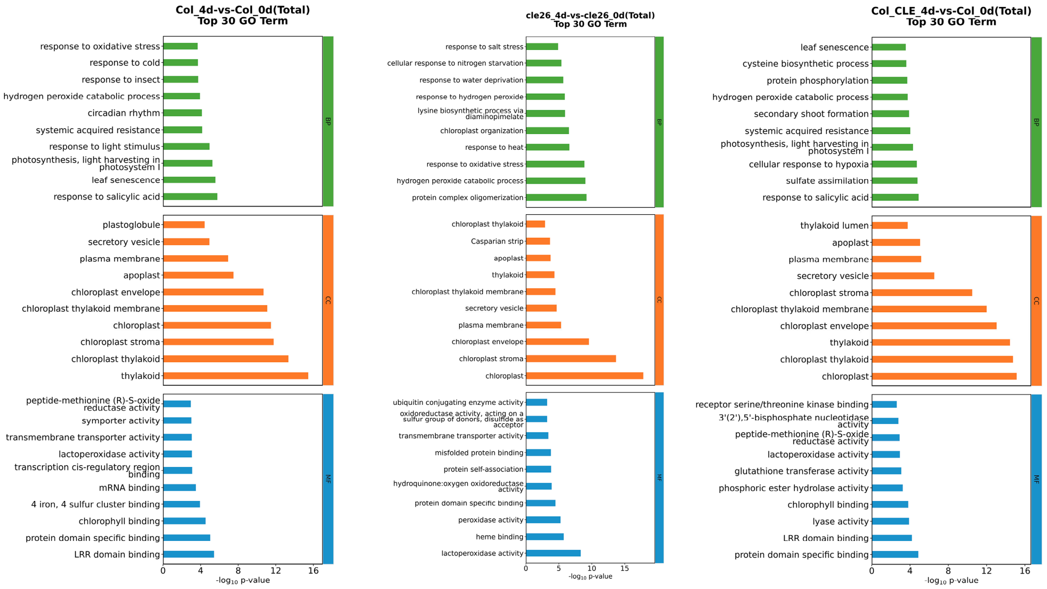

Figure S3 GO enrichment analysis of the DEGs in Col_4d vs Col_0d, cle26_4d vs cle26_0d, and Col_CLE_4d vs Col_0d.
